# Mediating roles of sleep quality and resilience in the relationships between chronotypes and mental health symptoms

**DOI:** 10.1038/s41598-024-56688-w

**Published:** 2024-03-11

**Authors:** Kean Poon, Mimi S. H. Ho, Alan P. L. Tai, Mei-Kei Leung, Meanne C. M. Chan, Way K. W. Lau

**Affiliations:** 1 School of Education, The University of New South Wales, Sydney, Australia; 2 Department of Special Education and Counselling, The Education University of Hong Kong, Hong Kong, China; 3 Department of Counselling and Psychology, Hong Kong Shue Yan University, Hong Kong, China; 4 Wofoo Joseph Lee Consulting and Counselling Psychology Research Centre, Lingnan University of Hong Kong, Room 213, LBY Building, 8 Castle Peak Road, Tuen Mun, Hong Kong China; 5 Department of Health Sciences, Hong Kong Metropolitan University, Room F1229, 12/F, 1 Sheung Shing Street, Ho Man Tin, Kowloon, Hong Kong, China

**Keywords:** Anxiety, Chronotypes, Depression, Resilience, Sleep quality, Sleep disorders, Quality of life, Human behaviour

## Abstract

Sleep and mental health are intrinsically intertwined, but not every individual with problems sleeping develops a mental health disorder. This study examined the association among chronotypes, resilience, sleep quality and mental health symptoms amongst otherwise healthy individuals. Two hundred adults (*M*_age_ = 27.75 ± 5.11, 68% female) with no previous diagnosis of mental illness were recruited and filled in a set of questionnaires measuring chronotypes, sleep quality, depression and anxiety symptoms. The findings from the path analysis showed that the morning type had a statistically significant direct effect on a range of sleep quality indices. These included better subjective sleep quality, shortened sleep latency, and fewer daytime dysfunctions, as well as a higher level of resilience. However, it did not significantly affect depression and anxiety symptoms. In addition, the morning type had statistically significant indirect effects on a higher level of resilience and fewer depression and anxiety symptoms through the mediating effect of sleep quality indices. Findings from this study support that morning type is associated with better resilience and psychological health, which is mediated through better sleep quality.

## Introduction

A good night’s sleep is essential to psychological well-being, but poor sleep is one of the key correlates of poor mental health^1^ and these intertwined struggles are becoming a rising public health concern^2^. Much of the literature documents the role of insomnia and mood disorders^3^, but there is also evidence that problems sleeping is associated with a host of mental health difficulties^1^. However, not every individual with problems sleeping is diagnosed with a mental health disorder, pointing to the possibility of resilience across both the sleep and psychological symptoms continuum. One parameter less examined in the context of mental health is chronotype, and this study sought to examine how chronotype may help explain individual differences in the sleep-mental health link.

Chronotype is defined as an internal clock that regulates daily rest and routines^4^. It is generally classified into morning, evening, and intermediate types, but the morning/evening preference is a continuum^5^. Morning type individuals naturally fall asleep and wake up early, while evening type individuals follow a later schedule. Individuals with intermediate type do not show a marked tendency for either pole^6,7^. In general, the three chronotypes follow a normal distribution with morning and evening types representing about 40% of the adult population (around 20% each), while the intermediate type make up the remaining 60%^4^.

In the Chinese population, Zhang et al.^8^ carried out a large-scale study to determine chronotypes using objective recordings with wearable devices. Chronotypes were assessed (*N* = 49,573) by the adjusted mid-point of sleep on free days. The percentages of Chinese people with morning, intermediate, and evening types were 26.76%, 58.59%, and 14.64%, respectively^8^. Liu et al.^9^ conducted a study to determine chronotypes using the Morningness-Eveningness Questionnaire (MEQ)^10^. A total of 8,395 participants across China were included, and the authors identified 11.3% of Chinese people as morning type, 70.6% as intermediate type, and 18.1% as evening type (*M*_age_ = 30.38 ± 11.47 years, 37.38% male). Although the measurements differ in these two studies, the percentages provide a general overview of chronotype distribution in the Chinese population.

### Chronotypes and mental health symptoms

The empirical evidence delineating the relationship between chronotypes and psychological well-being is extensive^11–13^. Notably, investigations conducted within diverse populations have illuminated various facets of this intricate association. For example, in a comprehensive population-based study encompassing 10,503 Finnish adults, the evening chronotype was found to be significantly associated with heightened levels of depressive symptoms or clinical diagnoses of depression^14^. Similarly, a study exploring the correlation between depressive symptoms and chronotypes among 1252 Japanese day workers and 1780 night shift workers revealed markedly elevated self-reported depressive scores among night shift workers compared to their daytime counterparts^15^. Furthermore, analyses from a sizable cohort comprising 1,944 individuals encompassing currently depressed patients, remitted patients, and healthy controls, underscored the prognostic significance of eveningness, demonstrating its predictive capacity for depressive symptoms and the onset of depressive episodes over a 12-month timeframe, irrespective of prior depressive episodes^16^. Collectively, these findings pointed to the direction that an evening chronotype can be a risk factor for the development of depression^17–20^.

While some research focused on the association between chronotypes and depression, emerging evidence suggests a link between chronotype and anxiety disorders. Specifically, Merikanto and Partonen^21^ observed a heightened susceptibility to anxiety symptoms among individuals characterized by an evening chronotype within a substantial sample of Finnish adults (N = 18,039). This association between eveningness and adverse mental health outcomes was found to be partially mediated by insufficient sleep^21^. Similarly, Lemoine et al.^22^ reported a higher prevalence of evening chronotypes among participants diagnosed with anxiety disorders.

Recent investigations have cast doubt on the one-way association between chronotype and susceptibility to mental health symptoms, suggesting that mental disorders may also influence chronotype preferences^23^. Furthermore, other studies have indicated that the link between chronotype and mental symptoms is not direct, emphasizing the impact of disrupted sleep patterns and cortisol secretion on mental health outcomes and revealing potential interactions between the circadian system and mood regulation^23,24^. Overall, the relationships between mental health and chronotype are far more complex than originally expected, highlighting the need for thorough investigation into the multifaceted nature of circadian activity and its effects on psychological well-being.

### Chronotypes and sleep quality

Chronotype has strong implications for sleep quality, with evening type reporting a range of sleep complaints, such as more frequent nightmares, shorter sleep duration, more frequent use of sleep medication, poorer sleep quality, and longer sleep onset latency than morning or intermediate types^25–28^. It is increasingly recognized that adequate sleep is required for mental health, and poor sleep quality is a risk factor in the development and maintenance of mood disorders^29,30^. Problematic sleep has also been shown to exacerbate symptom severity^31^. Nevertheless, studies on the impact of chronotype on mental outcomes and its relation to sleep quality are both limited and controversial. Previous longitudinal studies have reported that poor sleep quality was a predictor of depression^17^. In a healthy sample, Horne et al.^32^ observed a significant link between evening type and increased depressive symptoms, and this relationship was partially mediated by sleep quality. Levandovski et al.^33^ observed a relationship between sleep quality and depressive symptoms in addition to the eveningness-depression association. These findings provide preliminary evidence that mental health symptoms might be associated with chronotype, and sleep quality might mediate the relationship between chronotype and these symptoms.

However, several studies have suggested that chronotype might be a separate risk factor for mental health symptoms, beyond other sleep-related problems. For example, some studies have shown that sleep quality and sleep complaints did not mediate the relationship between evening type and depressive symptoms in healthy subjects^34,35^. Müller et al.^36^ reported that evening type and poorer subjective sleep quality were separately associated with higher depression severity in depressed patients. These studies might suggest that sleep quality alone is insufficient to fully explain the relationship between chronotype and depression, and additional factors are expected to play a role in better understanding the link between chronotype and mental health symptoms. Psychological resilience (hereafter called resilience) could be one of the important inter-players between chronotypes and depression.

### Resilience

Resilience is defined as the ability to adapt to change and the capacity to face disturbance^37^. It is a variable that consistently appears in the literature in relation to chronotype and mental health. Evidence shows that resilience works as a protective factor for a number of mental illnesses, including depression and anxiety^38^. Low levels of resilience are associated with a higher likelihood of developing a range of mental health problems^39,40^. Chronotypes are associated with resilience^41,42^. For instance, participants with morning type showed the highest resilience level, followed by the intermediate and evening types in a large sample of 1,922 student and adult participants^41^. Findings from another study of 1,094 Korean college students suggested that morning type was a significant positive predictor of resilience, even after controlling for age, sex, depressive and anxiety symptoms, and sleep quality^42^. Similar findings were reported in patients with major depressive disorder (MDD) after controlling for the influence of age, gender, length of education, economic status, onset age, and suicide attempt history^43^.

### Aim of the study

In summary, research studying the relationship between chronotype and mental health symptoms commonly investigates a singular relationship. However, the relationship between these constructs is complicated. Thus, it is crucial to explore the underlying factors that mediate the association in order to guide the development of future interventions to ease the strain posed to the health care system. The aim of this study was to understand the role of sleep quality and resilience in mediating the relationships between chronotype and mental health symptoms in a sample of healthy Chinese adults with no previous diagnosis of mental illness.

## Results

The demographic details are listed in Table 1. According to Buysse and colleagues^44^, subjects with a global PSQI score larger than five indicate an association with poor sleep quality. The mean global PSQI score of our participants was 6.26 ± 2.88, indicating that our participants had certain sleep problems. Twenty-seven (13.5%) participants were regarded as morning type, 130 (65%) participants were intermediate type, and 43 (21.5%) participants were evening type. The mean scores of DASS for depression and anxiety symptoms were 5.04 ± 4.65 and 5.66 ± 3.94, respectively, suggesting mild level of depression and moderate level of anxiety symptoms in our participants^45^. The Spearman’s rho correlation coefficient is shown in Table 2.

**Table 1:** Sample characteristics (N = 200).

Variables	Mean (SD)/N (%)
Age in years	27.75 (5.11)
Male:Female (% of Female)	64:136 (68%)
Education	
High school	23 (11.5%)
High diploma	22 (11.0%)
Bachelor’s degree	117 (58.5%)
Master’s degree	31 (15.5%)
Doctorate degree	7 (3.5%)
Consumption of caffeine beverages	
Never	45 (22.5%)
Once per week	34 (17.0%)
2–3 times per week	55 (27.5%)
4–5 times per week	27 (13.5%)
More than 5 times per week	39 (19.5%)
Pittsburgh Sleep Quality Index (PSQI)	
Global PSQI score	6.26 (2.88)
Subjective sleep quality	1.24 (0.70)
Sleep latency	1.03 (0.86)
Sleep duration	1.22 (0.91)
Habitual sleep efficiency	0.67 (1.01)
Sleep disturbance	1.12 (0.49)
Use of sleep medication	0.03 (0.17)
Daytime dysfunction	0.95 (0.77)
Morningness–Eveningness Questionnaire (MEQ)	
MEQ score	48.48 (9.00)
MEQ subtypes	
Morning type	27 (13.5%)
Intermediate type	130 (65.0%)
Evening type	43 (21.5%)
Connor–Davidson Resilience Scale (CD-RISC)	
CD-RISC score	64.42 (11.56)
Depression Anxiety and Stress Scale (DASS)	
Depressive symptoms score	5.04 (4.65)
Anxiety symptoms score	5.66 (3.94)

**Table 2:** Spearman’s rho correlation among all variables.

	1	2	3	4	5	6	7	8	9	10	11	12	13	14	15
1. MEQ score	**–**	**0.258**	− 0.201	− 0.161	− 0.082	− 0.054	− 0.105	0.079	− 0.128	− 0.122	− 0.173	0.086	0.078	0.168	− 0.057
2. CD-RISC		**–**	**− 0.245**	− 0.152	− 0.207	− 0.152	− 0.106	− 0.029	− 0.185	**− 0.356**	**− 0.458**	− 0.081	0.104	0.167	0.041
3. Subjective sleep quality*			**–**	**0.366**	**0.322**	**0.370**	**0.349**	− 0.008	**0.434**	**0.386**	**0.377**	− 0.073	− 0.018	− 0.139	0.002
4. Sleep latency*				**–**	0.019	**0.357**	**0.392**	0.092	**0.314**	**0.348**	**0.334**	− 0.055	− 0.138	− 0.114	− 0.131
5. Sleep duration*					**–**	0.170	0.074	− 0.012	0.125	− 0.010	0.030	− 0.124	− 0.027	− 0.143	0.010
6. Habitual sleep efficiency*						**–**	0.163	0.116	0.137	**0.289**	**0.279**	0.108	− 0.053	− 0.025	− 0.138
7. Sleep disturbance*							**–**	0.081	**0.344**	**0.356**	**0.259**	− 0.123	0.030	− 0.246	0.008
8. Use of sleep* medication								**–**	0.063	0.183	0.164	0.058	− 0.153	− 0.038	0.018
9. Daytime* dysfunction									**–**	**0.365**	**0.424**	− 0.107	− 0.012	0.048	0.084
10. DASS-anxiety symptoms										**–**	**0.761**	0.051	− 0.240	− 0.107	0.095
11. DASS-depression symptoms											**–**	− 0.032	**− 0.252**	− 0.151	0.021
12. Gender												**–**	− 0.146	0.125	− 0.008
13. Age													**–**	0.072	0.197
14. Education														**–**	0.045
15. Consumption of caffeine products															**–**

Numerical values represented Spearman’s rho correlation coefficient. Bonferroni corrected *p*-value = 0.00048 (0.05/105 comparisons). Significant correlations were bold. MEQ, Morningness–Eveningness Questionnaire; CD-RISC, Connor–Davidson Resilience Scale; DASS, Depression Anxiety and Stress Scale. Higher MEQ scores indicate a higher tendency towards the morning type. Higher scores in each of the Pittsburgh Sleep Quality Index (PSQI) components indicate greater problems in that particular aspect.

### Behavioral differences across chronotypes

Resilience level, sleep problems / quality, and the level of depression and anxiety symptoms were compared across the three different chronotypes in our samples. There was a significant difference in resilience level (χ^2^ = 16.462, *df* = 2, *p* < 0.001, Cohen’s *f* = 0.308) across the three chronotypes. Post-hoc analyses indicated that resilience level in intermediate type (64.47 ± 10.97, *p* < 0.005) or evening type (60.14 ± 12.69, *p* < 0.0005) was significantly lower than that of morning type (71.00 ± 9.53), whereas no significant differences were observed between intermediate type and evening type. On the other hand, no significant differences in the global PSQI score (χ^2^ = 3.995, *df* = 2, *p* = 0.136) and the level of depression (χ^2^ = 3.822, *df* = 2, *p* = 0.148), and anxiety symptoms (χ^2^ = 1.570, *df* = 2, *p* = 0.456) were observed.

### Path analysis

**Figure 1:**
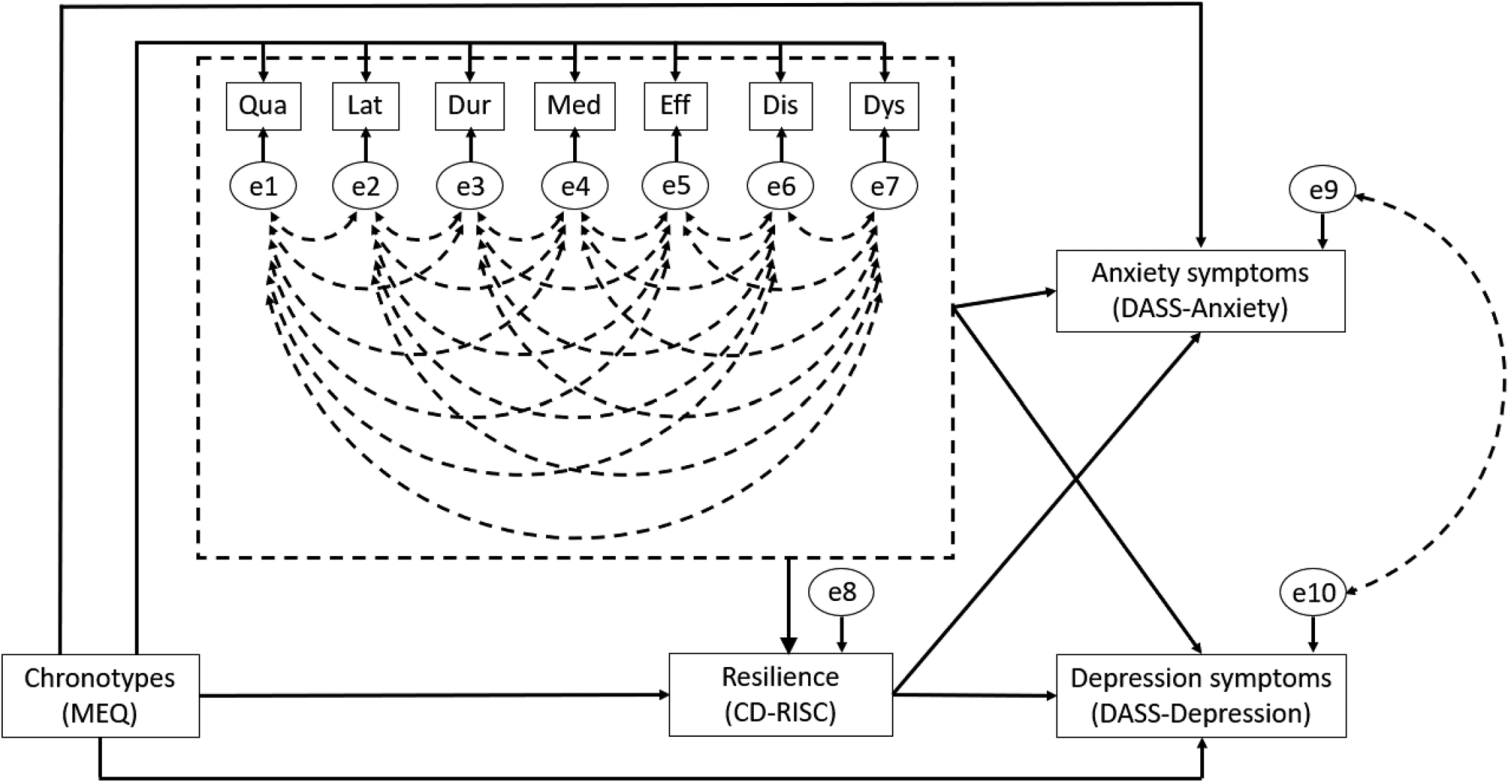
The finalized Pittsburgh Sleep Quality Index (PSQI) constraint model. Each exogenous variable includes an error term (e1-10). The effect of seven subscales of the PSQI on Connor-Davidson Resilience Scale (CD-RISC) was constrained to be equal, which is represented by the dotted line with an arrowhead. These subscales are subjective sleep quality (Qua), sleep latency (Lat), sleep duration (Dur), habitual sleep efficiency (Eff), sleep disturbance (Dis), use of sleep medication (Med), and daytime dysfunction (Dys). Covariations between errors are illustrated by dotted lines with double arrowheads. MEQ, Morningness–Eveningness Questionnaire; DASS, Depression Anxiety Stress Scale.

Chi-square statistics as well as the multiple mode fit indices indicated that the unconstraint model fitted our data. Chi-square difference tests further suggested that both the MEQ constraint model (Δ*df* = 6, Δχ^2^ = 12.397, *p* = 0.054) and the PSQI constraint model (Δ*df* = 6, Δχ^2^ = 4.232, *p* = 0.645) were significantly better than the unconstraint model. However, in the MEQ constraint model, some of the fit indices did not meet the cut-off criteria (i.e., TLI < 0.95, RMESA > 0.05, Table 3). We therefore considered this MEQ constraint model not fitting our data well. The proceeding analyses supported that the PSQI constraint model had the best fit to our data. Therefore, path analysis was conducted in this PSQI constraint model. The final model is illustrated in Fig. 1.

**Table 3:** Model comparison among the unconstraint and nested models.

Models	χ^2^ (*df*)	*p*-value^#^	NFI	CFI	TLI	SRMR	RMSEA	Δχ^2^	Δ*df*	*p*-value^##^
Unconstraint	0.833 (1)	0.369	0.999	1.000	1.019	0.010	< 0.001			
Equality constraint on sleep problems from MEQ	13.230 (7)	0.099	0.979	0.989	0.896	0.042	0.067	12.397	6	0.054
Equality constraint on resilience from sleep problems	**5.064 (7)**	**0.636**	**0.992**	**1.000**	**1.032**	**0.019**	** < 0.001**	**4.232**	**6**	**0.645**

CFI, Comparative fit index; *df*, degree of freedom; MEQ, Morningness–Eveningness Questionnaire; NFI, Normed fit index; RMSEA, Root-mean-square error of approximation; SRMR, Standardized root mean squared residual; TLI, Tucker–Lewis index. #*p*-values were obtained from Chi-square statistics (χ^2^) using Bollen-Stine bootstrapping method (5000 times). ##*p*-values were obtained from Chi-square difference tests compared with the freely estimated model. The effect of age was controlled in both the unconstraint and nested models. The final model was bold.

### Direct and indirect effects

Direct and indirect effect paths were estimated in the PSQI constraint model. Statistically significant direct effects of MEQ scores on subjective sleep quality (Unstandardized β = − 0.015, Bootstrap SE = 0.005, 95% CI = − 0.026 to − 0.005, *p* = 0.005), sleep latency (Unstandardized β = − 0.015, Bootstrap SE = 0.007, 95% CI = − 0.029 to − 0.002, *p* = 0.03), use of sleep medication (Unstandardized β = 0.002, Bootstrap SE = 0.001, 95% CI = 0.000 to 0.006, p = 0.03), and daytime dysfunction (Unstandardized β = − 0.013, Bootstrap SE = 0.006, 95% CI = − 0.025 to − 0.001, *p* = 0.034) in the subscales of PSQI were observed. In addition, a statistically significant direct effect of the MEQ scores on CD-RISC (Unstandardized β = 0.218, Bootstrap SE = 0.088, 95% CI = 0.039 to 0.390, *p* = 0.013) was observed, whereas the direct effects of the MEQ scores on the level of depression and anxiety symptoms were non-significant. Since the effect of the PSQI subscales on CD-RISC was constraint to be equal, the unstandardized β across the seven subscales of PSQI on CD-RISC was the same, which was found to significantly predict CD-RISC (Unstandardized β = − 1.064, Bootstrap SE = 0.280, 95% CI = − 1.622 to − 0.522, *p* < 0.001).

On the other hand, differential direct effects of the PSQI subscales on the level of depression and anxiety symptoms were found. Sleep duration (Unstandardized β = − 1.035, Bootstrap SE = 0.264, 95% CI = − 1.529 to − 0.503, *p* < 0.001), habitual sleep efficiency (Unstandardized β = 0.553, Bootstrap SE = 0.215, 95% CI = 0.137 to 0.965, *p* = 0.011), sleep disturbance (Unstandardized β = 1.501, Bootstrap SE = 0.490, 95% CI = 0.576 to 2.500, *p* = 0.003), use of sleep medication (Unstandardized β = 3.029, Bootstrap SE = 1.183, 95% CI = 0.866 to 5.660, *p* = 0.011), and daytime dysfunction (Unstandardized β = 0.996, Bootstrap SE = 0.322, 95% CI = 0.389 to 1.642,* p* = 0.001) were the only significant predictors in the subscales of the PSQI for predicting the level of anxiety symptoms.

For predicting the level of depression symptoms, only sleep duration (Unstandardized β = − 1.017, Bootstrap SE = 0.288, 95% CI = − 1.576 to − 0.450, *p* = 0.001) and daytime dysfunction (Unstandardized β = 1.460, Bootstrap SE = 0.373, 95% CI = 0.736 to 2.216, *p* < 0.001) were the significant predictors in the subscales of PSQI. Significant direct effect of CD-RISC was observed in predicting the level of anxiety symptoms (Unstandardized β = − 0.093, Bootstrap SE = 0.020, 95% CI = − 0.133 to − 0.052, *p* < 0.001) and depression symptoms (Unstandardized β = − 0.156, Bootstrap SE = 0.023, 95% CI = − 0.200 to − 0.111, *p* < 0.001) (see Table 4).

**Table 4:** Direct effects in the finalized constraint model.

Independent variables	Dependent variables	Unstandardized β	Bootstrap SE	95% CI	*p*-value
MEQ	Subjective sleep quality	**− 0.015**	**0.005**	**− .026 to − 0.005**	**0.005**
	Sleep latency	**− 0.015**	**0.007**	**− 0.029 to − 0.002**	**0.030**
	Sleep duration	− 0.008	0.008	− 0.023 to 0.008	0.339
	Habitual sleep efficiency	− 0.003	0.009	− 0.019 to 0.014	0.744
	Sleep disturbance	− 0.005	0.004	− 0.012 to 0.002	0.207
	Use of sleep medication	**0.002**	**0.001**	**0.000 to 0.006**	**0.030**
	Daytime dysfunction	**− 0.013**	**0.006**	**− 0.025 to − 0.001**	**0.034**
	CD-RISC	**0.218**	**0.088**	**0.039 to 0.390**	**0.013**
	DASS-anxiety symptoms	0.021	0.025	− 0.025 to 0.072	0.339
	DASS-depression symptoms	0.015	0.029	− 0.041 to 0.073	0.545
PSQI subscales	CD-RISC	**− 1.064**	**0.280**	**− 1.622 to − 0.522**	** < 0.001**
Subjective sleep quality	DASS-anxiety symptoms	**1.303**	**0.376**	**0.554 to 2.025**	** < 0.001**
	DASS-depression symptoms	**1.390**	**0.487**	**0.435 to 2.231**	**0.005**
Sleep latency	DASS-anxiety symptoms	0.066	0.285	− 0.478 to 0.635	0.816
	DASS-depression symptoms	0.091	0.338	− 0.544 to 0.771	0.777
Sleep duration	DASS-anxiety symptoms	− **1.035**	**0.264**	− **1.529 to **− **0.503**	** < 0.001**
	DASS-depression symptoms	− **1.017**	**0.288**	− **1.576 to **− **0.450**	**0.001**
Habitual sleep efficiency	DASS-anxiety symptoms	**0.553**	**0.215**	**0.137 to 0.965**	**0.011**
	DASS-depression symptoms	0.544	0.289	− 0.028 to 1.097	0.067
Sleep disturbance	DASS-anxiety symptoms	**1.501**	**0.490**	**0.576 to 2.500**	**0.003**
	DASS-depression symptoms	0.949	0.606	− 0.243 to 2.148	0.113
Use of sleep medication	DASS-anxiety symptoms	**3.029**	**1.183**	**0.866 to 5.660**	**0.011**
	DASS-depression symptoms	2.183	1.873	− 1.126 to 6.211	0.208
Daytime dysfunction	DASS-anxiety symptoms	**0.996**	**0.322**	**0.389 to 1.642**	**0.001**
	DASS-depression symptoms	**1.460**	**0.373**	**0.736 to 2.216**	** < 0.001**
CD-RISC	DASS-anxiety symptoms	− **0.093**	**0.020**	− **0.133 to **− **0.052**	** < 0.001**
	DASS-depression symptoms	− **0.156**	**0.023**	− **0.200 to **− **0.111**	** < 0.001**

CI, Confidence interval; SE, Standard errors; MEQ, Morningness–Eveningness Questionnaire; CD-RISC, Connor–Davidson Resilience Scale; DASS, Depression Anxiety and Stress Scale; PSQI, Pittsburgh Sleep Quality Index. Significant paths were bold.

For the indirect paths, we found that MEQ scores had a significant indirect effect on CD-RISC (Unstandardized β = 0.060, Bootstrap SE = 0.029, 95% CI = 0.015 to 0.135, *p* = 0.006), the level of anxiety symptoms (Unstandardized β = − 0.053, Bootstrap SE = 0.024, 95% CI = − 0.107 to − 0.011, *p* = 0.01), and depression symptoms (Unstandardized β = − 0.078, Bootstrap SE = 0.029, 95% CI = − 0.143 to − 0.029, *p* = 0.003). On the other hand, the PSQI subscales had an overall significant indirect effect on the level of anxiety symptoms (Unstandardized β = 0.166, Bootstrap SE = 0.053, 95% CI = 0.076 to 0.286, *p* < 0.001) and depression symptoms (Unstandardized β = 0.166, Bootstrap SE = 0.053, 95% CI = 0.076 to 0.286, *p* < 0.001) (See Table 5).

**Table 5:** Indirect effects in the finalized constraint model.

Independent variables	Dependent variables	Unstandardized β	Bootstrap SE	95% CI	*p*-value
MEQ	CD-RISC	**0.060**	**0.029**	**0.015 to 0.135**	**0.006**
	DASS-anxiety symptoms	**− 0.053**	**0.024**	**− 0.107 to − 0.011**	**0.010**
	DASS-depression symptoms	**− 0.078**	**0.029**	**− 0.143 to − 0.029**	**0.003**
PSQI subscales	DASS-anxiety symptoms	**0.099**	**0.034**	**0.043 to 0.177**	** < 0.001**
	DASS-depression symptoms	**0.166**	**0.053**	**0.076 to 0.286**	** < 0.001**

CI, Confidence interval; SE, Standard errors; MEQ, Morningness–Eveningness Questionnaire; CD-RISC, Connor–Davidson Resilience Scale; DASS, Depression Anxiety and Stress Scale; PSQI, Pittsburgh Sleep Quality Index. Significant paths were bold.

## Discussion

Sleep struggles and mental health difficulties are intrinsically intertwined and involve multiple pathways. Adding to literature that has predominantly focused on insomnia and disorders, this study examined chronotypes and mental symptoms amongst otherwise healthy individuals, as well as the possible mechanisms of resilience and sleep quality.

The current study has first explored the behavioral differences across chronotypes. Similar to the distribution/pattern reported by Zhang et al.^8^, the majority of our participants reported being of the intermediate type (65%), followed by the evening type (21.5%), and morning type (13.5%). Comparing resilience level, sleep quality, and psychological symptoms, the morning type demonstrated statistically significant higher resilience levels than the other two chronotypes; no other differences were however identified. The effect size of such difference was considered medium based on Cohen’s *f* statistics. Moreover, it is surprising to note that, although our participants had no previous record of mental illness, they showed generally elevated psychological symptoms and poor sleep quality compared to international norms^46^. Specifically, on average, they demonstrated mild levels of depression and moderate levels of anxiety symptoms, echoing a recent study using a telephone survey in Hong Kong, which reported that highly stressed working environments and long working hours are conducive to poor sleep quality and reduced mental well-being^47^. Prevalence estimates of sleep quality in typical Western populations are well documented, but data for Chinese adults are comparatively scarce^48^. The current study provides additional evidence to support the high level and prevalence of negative psychological symptoms and poor sleep quality.

To understand the relationships between chronotypes and mental symptoms, our study suggested a new perspective and identified a complete mediation model between the two constructs. In other words, chronotypes have no direct effect on either level of depression or anxiety symptoms when controlling for the impact of sleep quality and resilience. Specifically, path analysis suggested that the relationships among these domains are unique and complex. In exploring the mediating role of sleep quality, the direct effects of chronotype are observed in some of the indices of sleep quality, such as subjective sleep quality, sleep latency, use of sleep medication, and daytime dysfunction. However, the effect size of these direct effects was considered small (unstandardized *β* < 0.1 in magnitude), the practical significance of the effect of chronotype on sleep quality remains questionable. These findings suggest that the propensity for an individual to sleep at a particular time may have minimal influence on various sleep parameters including sleep quality. It remains unclear whether circadian disruption is more severe in the evening type resulting in more sleep disturbance. This requires future studies to further confirm. In addition, caution must be taken for the interpretation of our results in the use of sleep medication. It is because there were only six participants rated 1 in this aspect, while the rest of the 194 participants indicated that they did not use sleep medication at all. Among the six participants who rated 1 in that area, five of them were in the intermediate-type and one of them was in the morning-type. These findings corroborate previous research that participants of the evening type were reported to have longer sleep latency, poorer sleep quality, and greater daytime dysfunction than participants of the morning or immediate types^49^. This might be due to individual differences stemming from their lifestyle preferences; there is a tendency for evening types to have irregular sleep habits or erratic sleep–wake schedules. Evening types were correlated with later bedtime and wake-up time on weekends, shorter time in bed during the weeks, and longer weekend time in bed^50^. It has also been reported that participants with evening types tend to consume more alcohol, caffeine, and tobacco, resulting in more frequent daytime sleepiness than participants with morning types^51,52^.

Direct effects of sleep quality on depression and anxiety symptoms are also observed, using parameters such as sleep duration, habitual sleep efficiency, sleep disturbance, use of sleep medication, and daytime dysfunction, all of which are associated with anxiety symptoms; however, only sleep duration and daytime dysfunction are significant in predicting levels of depression. It is suggested that these parameters related to subjective sleep difficulties may have unique implications for the mechanism delineating between chronotype and mental health symptoms. Our findings cohere with previous research that daytime dysfunction and shorter sleep duration were positively associated with depression^53^. It is possible that daytime dysfunction could stem from insomnia, a major symptom of depression^54^. Individuals sleeping for a short period of time may lead to insufficient rest and a greater perceived stress severity, which has been reported as a risk factor for depression^55,56^. In fact, an indicator of a healthy lifestyle could be good sleep conditions and mental well-being.

Our findings also discovered the mediating role of resilience in explaining the relationship between chronotype and mental health. While the role of resilience remains unclear, other recent studies found that sleep disturbance^57^, sleep quality^58,59^, social support^60,61^, and rumination^62^ were significant mediators of the relationships between chronotype and depression and anxiety symptoms. Nevertheless, our study found that participants with morning type had higher resilience than participants with evening type, confirming previous research showing that morning types demonstrate stronger resilience than evening types among healthy adults^41^.

It appears that chronotype affects resilience; this may be due to disrupted daily routines^63,64^ and circadian rhythms^65,66^. Strong resilience requires adaptability to work in social situations as well as psychological and physical conditions^37^. It is clear that individuals with high resilience are able to cope with challenging situations, and resilience is demonstrated to be protective against depression in college students^67,68^. A Korean study^42^ found that morning chronotype and longer daytime exposure to sunlight between 10:00 and 15:00 were significantly associated with greater resilience. In contrast, individuals of evening chronotype are more likely to experience social jetlag caused by desynchronized biological and social clocks, resulting in lower resilience^41,69^. In turn, sleep problems and reduced resilience are more likely to facilitate the development of mental health issues. This is in accordance with a meta-analytical review that resilience was negatively correlated with indicators of ill-being (e.g., anxiety, depression, negative affect) and positively correlated with indicators of well-being (e.g., life satisfaction, positive affect)^70^.

Another possible explanation is that the contribution of family, friends, and colleagues to social support may help explain the relationship between evening chronotype and depression^61^. It has been shown that morning chronotypes have a higher level of social support than evening chronotypes. Individuals who belong to the evening chronotype have conflicts in their social lives, with feelings of loneliness and low support from others^60^. While evening chronotypes may have adequate social support, they may not be aware or willing to acknowledge it due to negative cognitive bias^60^. However, prior to concluding these findings, future research is now required to determine whether social support helps explain the relationship between chronotypes and mental health in Chinese healthy adults.

Statistically, we demonstrated significant direct paths linking the three constructs, whereas the effect size of chronotype on subjective sleep quality was rather small. Similarly, sleep quality had statistically significant mediation effect on the relationship between chronotype and resilience, although the effect was relatively small. The magnitude of direct and indirect effects should be re-evaluated in a bigger sample with an equal number of participants in each chronotype. Also, circadian disruption should be considered as an additional parameter in future mediation models. Nevertheless, sleep is an important brain function for memory consolidation, brain reactivity, and emotional regulation^71,72^. A 60% increase in amygdala activity in response to emotional stimuli was identified under conditions of poor sleep quality, which leads to reduced resilience^73^. Our findings are novel, as no previous studies have examined the role of sleep quality in explaining the relationship between chronotype and resilience. It is crucial to reaffirm the importance of sleep quality to improve resilience and mental health, as previous studies found that psychological well-being problems were less likely to occur with normal sleep quality^74^. People with better sleep quality also had higher resilience^75^.

## Limitations and conclusions

The current study has some limitations that should be taken into consideration and points to suggestions for further research. This study employed a cross-sectional approach which could not make causal inferences. A longitudinal approach is needed to verify the mediation model presented in this study. The measurements of sleep quality, depression and anxiety symptoms were self-reported using questionnaires. It is unknown whether the participants actually had irregular patterns. A recent study^76^ revealed a U-shaped association between sleep duration and depression: Both insufficient sleep (< 8 h) and excessive sleep (> 8 h) increase the risk of depression. Researchers may wish to consider using more accurate wearable devices (e.g., actigraphy, mental health technologies such as smartphone applications) for an objective measure of sleep quality and using an observational approach to identify depression and anxiety symptoms^77^. It is also worth noting that the participants in this study had a higher education background with approximately 78% of them holding a bachelor’s degree or above. Our findings may not be generalizable to those with a lower education background. Finally, this study only used age as the controlling variable—there might be other confounding variables that have not been controlled for due to data availability (e.g., medication usage, occupation, socioeconomic status).

Both sleep and mental health are critical aspects of daily life, highlighting the relevance of this complex relationship and the pressing need to unravel it further. This study not only shows that chronotype and sleep quality are associated with depression and anxiety symptoms, but also unearths the mechanistic roles of sleep quality and resilience in the relationship between chronotype and mental health. Our findings suggest that cultivating resilience while improving sleep hygiene with consideration of different chronotypes may be a viable intervention opportunity for improving well-being.

## Methods

### Participants

Two hundred adults (*M*_age_ = 27.75 ± 5.11; 136 females and 64 males) participated in this study. The demographic details are listed in Table 1. Snowball sampling method was adopted. Participants were recruited via social media (e.g., WhatsApp). Inclusion criteria were mentally healthy adults without a history of psychiatric or psychological disorders; substance and/or alcohol abuse that might influence their sleeping quality and/or mental health status. Participants were required to declare in the online form if they had the abovementioned issues before joining the study. Data collection was completed in March 2022, and the data analysis was completed in January 2023. The study procedure was approved by the Research Ethics Committee of The Education University of Hong Kong (EdUHK; reference number: 2018-2019-0117-01). All experiments were performed in accordance with relevant guidelines and regulations.

### Study design

All participants were well informed of the study aims and procedure through an online information sheet, they provided an online informed consent before participating in the study. Participants then filled in a set of online questionnaires including Morningness–Eveningness Questionnaire (MEQ)^78^, The Pittsburgh Sleep Quality Index (PSQI)^79^, Connor-Davidson Resilience Scale (CD-RISC)^80^, Depression Anxiety Stress Scales (DASS)^81^, and sociodemographic details.

### Instruments

#### Morningness–Eveniangness Questionnaire (MEQ)

The MEQ determines the chronotype (i.e., morning type, intermediate type, or evening type) based on participants’ preferred bedtime, get-up time, tiredness in the morning, peak performance times, and a global self-assessment item. It consists of 19 items. Participants are classified as morning type (score: 59–86), intermediate type (score: 42–58). and evening type (score: 16–41), depending on their scores^78^.

#### The Pittsburgh Sleep Quality Index (PSQI)

The PSQI assesses seven dimensions of sleep quality over the past month: subjective sleep quality, sleep latency, sleep duration, habitual sleep efficiency, sleep disturbance, use of sleep medication, and daytime dysfunction^79^. A global PSQI score larger than 5 is regarded as having sleep problems. Higher scores indicate more difficulties in sleep.

#### Connor–Davidson Resilience Scale (CD-RISC)

The CD-RISC measures the overall perceived resilience of oneself^80^. It has been reported as a reliable tool to assess resilience^82^. It consists of 25 items rated on a 5-point Likert scale from 0 (*not true at all*) to 4 (*true nearly all the time*). Higher scores represent higher resilience.

#### Depression, Anxiety and Stress Scale (DASS)

The DASS measures the affective states of depression, anxiety, stress, and general psychological distress^81^. It consists of 21 items with 7 items in each subscale. The depression (DASS-depression, 7 items) and anxiety (DASS-anxiety, 7 items) subscales were used in this study. Participants were asked to rate from 0 (*did not apply to me at all*) to 3 (*applied to me very much*). Higher scores indicate more severe symptoms of that area in the past month.

### Statistical analysis

The univariate normality of the data was tested by the Shapiro–Wilk analysis. All outcome measures were non-normally distributed (*ps* < 0.05). Non-parametric analyses e.g., Spearman’s rho correlation, Mann–Whitney U test, Kruskal–Wallis test, and bootstrapping (5000 times) technique were therefore adopted, where appropriate.

Spearman’s rho correlation was performed to test the association among all the variables. Bonferroni correction method was adopted to control for the effect of multiple comparisons, the corrected *p*-value is defined as 0.00048 (0.05/105 comparisons).

To determine the group-differences in resilience, sleep problems and mental health symptoms across participants with different chronotypes, multiple independent Kruskal–Wallis tests were performed. Scores of CD-RISC, global PSQI, DASS-depression, and DASS-anxiety were compared across morning type, intermediate type and evening type. For significant findings, post-hoc Mann–Whitney U tests were performed to determine the significant mean difference between morning-to-intermediate, morning-to-evening, and intermediate-to-evening types. Bonferroni correction method was adopted to control for the effect of multiple comparisons, the corrected *p*-value is defined as 0.016 (0.05/3 comparisons).

To examine the associations among chronotypes, sleep problems, resilience, anxiety, and depression symptoms, path analysis was performed using maximum likelihood estimation. All variables were treated as continuous observed variables. The MEQ score was the independent variable, the depression and anxiety symptoms were the dependent variables. The PSQI subscales including subjective sleep quality, sleep latency, sleep duration, habitual sleep efficiency, sleep disturbance, use of sleep medication, and daytime dysfunction, were set as the mediators between MEQ and CD-RISC; CD-RISC score was set as the mediator between the subscales of PSQI and the dependent variables. Direct effects of MEQ on CD-RISC, depression, and anxiety symptoms were included. Finally, direct effects of the PSQI subscales on depression and anxiety symptoms were also included. The residuals from each of the PSQI subscale were covaried. In addition, the residuals from depression and anxiety symptoms were covaried. The effect of age was controlled because it was significantly associated with depressive symptoms (Table 2).

Since all the outcome variables were not normally distributed, Bollen-Stine bootstrapping (5000 times) was performed to confirm the correctness of the models^83^. A *p*-value of > 0.05 supports the null hypothesis that the model fits the data. To further determine the model fit, the recommended cut-off values of multiple model fit indices (i.e., Chi-square (χ2)/degree of freedom (*df*): < 3, normed fit index (NFI), comparative fit index (CFI) and Tucker-Lewis index (TLI): > 0.95, and root mean square error of approximation (RMSEA) and standardized root mean square residual (SRMR): < 0.05) were adopted^84^. Three models were constructed in total for comparison, including an unconstraint model and two nested models. To examine the effect of MEQ on the subscales of the PSQI, a nested model was constructed by constraining the effect of the MEQ on subscales of the PSQI to be equal (MEQ constraint model), while all other parameters were freely estimated. To determine the differential effect of sleep problems on resilience, another nested model was constructed by constraining the effect of subscales of the PSQI on CD-RISC to be equal (PSQI constraint model), while all other parameters were freely estimated. The nested models were compared with the unconstraint model using Chi-square difference tests. A *p*-value of > 0.05 supports the null hypothesis that the nested model(s) is/are better than the unconstraint model. The unstandardized coefficients including direct and indirect effects within the model were estimated using a bias-corrected bootstrap technique (5000 times).

Statistical analyses were conducted using the Statistical Package for Social Science (IBM SPSS v24) and Analysis of Moment Structures (IBM AMOS v26). A *p*-value of < 0.05 is considered statistically significant unless otherwise specified.

## Data Availability

The datasets generated during and/or analyzed during the current study are available from the corresponding author on reasonable request.
